# Site-Selective Artificial Ribonucleases: Renaissance of Oligonucleotide Conjugates for Irreversible Cleavage of RNA Sequences

**DOI:** 10.3390/molecules26061732

**Published:** 2021-03-19

**Authors:** Yaroslav Staroseletz, Svetlana Gaponova, Olga Patutina, Elena Bichenkova, Bahareh Amirloo, Thomas Heyman, Daria Chiglintseva, Marina Zenkova

**Affiliations:** 1 Laboratory of Nucleic Acids Biochemistry, Institute of Chemical Biology and Fundamental Medicine SB RAS, Lavrentiev’s Ave. 8, 630090 Novosibirsk, Russia; staroselec@ngs.ru (Y.S.); sveta-mira@yandex.ru (S.G.); patutina@niboch.nsc.ru (O.P.); dashachiglintseva@gmail.com (D.C.); 2 School of Health Sciences, Faculty of Biology, Medicine and Health, University of Manchester, Oxford Rd., Manchester M13 9PT, UK; Elena.V.Bichenkova@manchester.ac.uk (E.B.); bahareh.amirloo@manchester.ac.uk (B.A.); thomas.heyman@manchester.ac.uk (T.H.)

**Keywords:** artificial ribonuclease, oligonucleotide-peptide conjugate, RNA cleavage, neocuproine, Tris(2-aminobenzimidazole), PNAzyme, miRNase

## Abstract

RNA-targeting therapeutics require highly efficient sequence-specific devices capable of RNA irreversible degradation in vivo. The most developed methods of sequence-specific RNA cleavage, such as siRNA or antisense oligonucleotides (ASO), are currently based on recruitment of either intracellular multi-protein complexes or enzymes, leaving alternative approaches (e.g., ribozymes and DNAzymes) far behind. Recently, site-selective artificial ribonucleases combining the oligonucleotide recognition motifs (or their structural analogues) and catalytically active groups in a single molecular scaffold have been proven to be a great competitor to siRNA and ASO. Using the most efficient catalytic groups, utilising both metal ion-dependent (Cu(II)-2,9-dimethylphenanthroline) and metal ion-free (Tris(2-aminobenzimidazole)) on the one hand and PNA as an RNA recognising oligonucleotide on the other, allowed site-selective artificial RNases to be created with half-lives of 0.5–1 h. Artificial RNases based on the catalytic peptide [(ArgLeu)_2_Gly]_2_ were able to take progress a step further by demonstrating an ability to cleave miRNA-21 in tumour cells and provide a significant reduction of tumour growth in mice.

## 1. Introduction

### 1.1. From Antisense Oligonucleotides to Site-Selective Ribonucleases

The idea of sequence-specific inactivation of pathogenic RNA with the use of antisense oligonucleotides was first proposed several decades ago [1,2,3] and initially performed in a cell-free system [4], followed by further experiments on the inhibition of Rous sarcoma virus replication and cell transformation [5,6]. Although the concept of sequence-specific inhibition of RNA has been confirmed experimentally, a number of barriers remained which needed to be solved before this approach could be translated into safe and effective therapeutics. One of the main barriers in the application of antisense oligonucleotide technology was the rapid degradation of DNA-based oligonucleotides in cells by nucleases, which could be addressed by the use of nuclease-resistant DNA or RNA analogues. Phosphorothioate [7], 2′-OMe [8], peptide nucleic acid (PNA) [9] and mesyl (methanesulphonyl) phosphoramidate [10] modifications are recognised as the most successful and widely used oligonucleotide derivatives. Significant progress achieved in the development of ASO is evident from the fact that five antisense oligonucleotide-based therapeutics have been approved by the FDA [11,12,13,14,15]. Historically, ASO technology was the first and therefore the most elaborated approach for sequence-selective RNA-scission; however, this method is not the only one. siRNA [16], and, to a lesser extent, ribozymes [17], DNAzymes [18,19] and CRISPR-Cas [20] represent viable alternatives for RNA targeting to ASO. Recently discovered artificial ribonucleases (aRNases) [21] represent a distinctive class of catalytically active molecules that are capable of cleaving RNA sequences without the recruitment of endogenous (e.g., enzymes) or exogenous (e.g., metal ions) factors. Acting in truly catalytic mode, ss-aRNases demonstrate utterly effective degradation of biologically relevant targets in vitro and in vivo, hence, approving oneself as a new class of tumour-related RNA inhibitors. 

This review contains consecutive descriptions of different aspects of ss-aRNase development from chemical issues such as design and synthesis to the essential biological characteristics including specificity, selectivity and efficiency of action in vitro and in vivo. With this in mind, the manuscript may be considered as a single text or as individual chapters drawing attention of the broad audience: from specialists in organic synthesis to molecular biologists and biochemists. For the convenience, the main characteristics of reviewed ss-aRNases are summarized in Table 1. 

### 1.2. Initial Stage of aRNase Development: Screening of Chemical Groups and General Structures

Antisense oligonucleotides silence RNA targets either by acting as steric blocks of functionally significant regions, or as guide sequences for recruited RNase H. The attachment of molecular scissors of various chemical natures to the oligonucleotide allows its gene-silencing properties to be improved via the irreversible destruction of RNA chains. Such approaches can also offer an opportunity for the functional manipulation of RNA. The realisation of this concept resulted in the appearance of a variety of aRNases.

An inherent feature of site-selective artificial ribonucleases (ss-aRNases), which are conjugates of an oligonucleotide and a catalytic moiety, is their capability of RNA sequence recognition, which is provided by the oligonucleotide domain, and cleavage of phosphodiester linkages, which is mediated by the catalytic domain. Nonspecific aRNases lacking the RNA-recognising motifs are beyond the scope of this review. Since 1994, when the first ss-aRNases were created [22,23,24], a great variety of chemical constructs have been employed and tested as catalytic domains for aRNases. The initial stage of aRNases development was thoroughly analysed in the book “Artificial Nucleases” [25] and in a comprehensive review published by Lönnberg’s group [21]. Already then, the main directions, challenges and peculiarities of this field were identified. 

All varieties of chemical moieties used as a catalytic domain for ss-aRNases fall into two main categories, i.e., metal ion-dependent and metal ion-independent chemical constructs. In turn, the first group can also be divided into two subgroups: lanthanide ion chelates and Cu^2+^ and Zn^2+^ chelates. Although the pioneering studies demonstrated a higher efficiency of metal-ion dependent aRNases [22,23,24], they tend to suffer from metal leakage or loss, as well as metal ion exchange reactions under intracellular conditions. Therefore, metal-free cleaving constructs started attracting increasing attention as potentially less toxic and more controllable catalysts. Although metal free aRNases are less efficient than metal-dependent ss-aRNases so far [26], their catalytic potential might be considerably improved by optimising mutual orientations of the key players in RNA catalysis. 

The cleaving domains of ss-aRNases tend to mimic (to some extent) the catalytic centre of natural enzymes (e.g., RNase A) which contains amino acid residues with imidazolic, guanidinium and/or amine functional groups such as histidine, arginine or lysine. With this in mind, the recruitment of peptides as RNA-cleaving domains in ss-aRNases was predictable. The ribonuclease activity of several peptides was confirmed in a series of early works [27,28]. In particular, peptides (20-mers or longer) with regularly alternating hydrophobic and basic amino acids showed ribonuclease activity; the most active were peptides with alternating leucine and arginine residues. This sparked the idea of using the [(ArgLeu)_4_Gly] peptide as a cleaving construct during the initial stage of ss-aRNases development while targeting tRNA^Lys^ [29]. 

**Figure 1:**
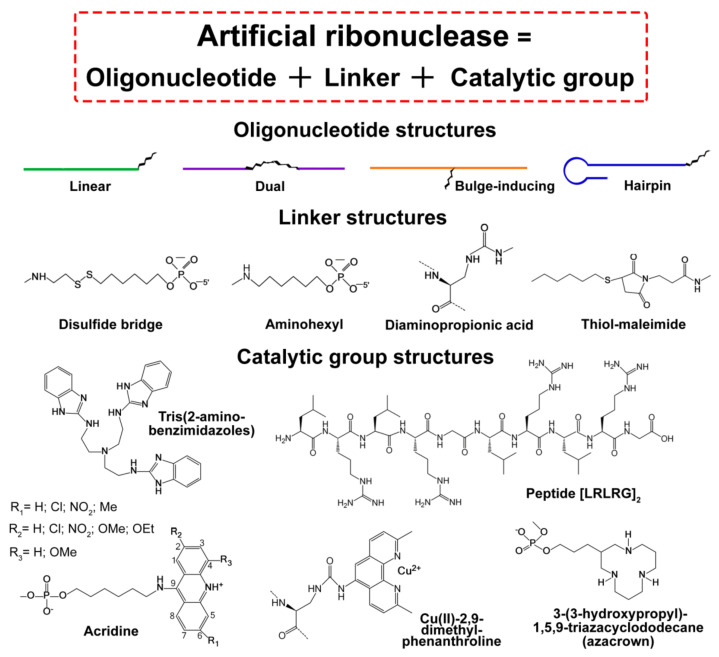
General design concept of currently established ss-aRNase, summarising the key structural features of the oligonucleotide recognition motifs (top), linkers (middle) and catalytic domains (bottom).

In the last two decades, the development of ss-aRNases for potential use in either therapy or RNA functional analysis was a “roller coaster”, with many failures, but also with some undeniable success, which allowed their benefits and advantages over the other established RNA-targeting approaches to be demonstrated. Here, we tried to track the path of the development of ss-aRNases in recent years by paying particular attention to the undeniably successful ss-aRNases, both metal-ion dependent and metal-ion free, while those structural variants which failed either during the development phase or application were excluded from our consideration. Figure 1 illustrates a general concept in the design of currently established ss-aRNases and gives some examples of the key structural components, including the oligonucleotide recognition motif (top), linker (middle) and catalytic domain (bottom). In terms of RNA cleaving constructs, the most actively used groups were trisbenzimidazole [26,30,31,32], imidazole [33] and the peptide [(ArgLeu)_2_Gly]_2_ [34,35,36], which represent metal ion-independent catalysts (Figure 1, Table 1). Another efficient catalytic group was dimethylphenanthroline, which chelates either Cu^2+^ or Zn^2+^ (Figure 1, Table 1), and thus represents metal-dependent catalysts [37,38,39,40]. Alongside these, there are several rarely used groups (acridine and azacrown) which also deserve some attention. 

The structural properties of the ss-aRNases and the nature of the oligonucleotide recognition motifs represent other factors underpinning their success. DNA oligonucleotides were used as recognition motifs in many initial in vitro studies of aRNases, but in vivo practice requires the employment of their nuclease-resistant chemical analogues, which are more stable in a cellular environment. Gapmer oligonucleotides consisting of a central stretch of DNA or phosphorothioate DNA monomers which enable one to recruit RNase H flanked with modified nucleotides such as 2′-*O*-methyl or 2′-*O*-methoxyethyl brining in nuclease resistance [8,9]. In order to increase substrate affinities of ss-aRNases some DNA residues can be replaced with diverse type of monomers such as locked nucleic acids (LNA) to form mixmers [32]. In that respect, ss-aRNases are similar to ASO, and many oligonucleotide analogues, which were initially introduced and tested as ASO, were later successfully used as an RNA-recognition domain within ss-aRNases. Amongst such analogues, PNAs deserve special attention, as they were used as structural elements within two highly successful series of aRNases; the first was based on the trisbenzimidazole catalyst [26,30,31,32], and the second was based on the dimethylphenanthroline cleaving group [37,38,39,40]. By switching from DNA oligonucleotides to the PNA backbone, not only was a considerable increase in the nuclease resistance of the ss-aRNase in vivo achieved, but the binding affinity and cleavage activity of the conjugate was also improved [26]. 

In terms of the structural organisation of such chemical ribonucleases, the first generation of aRNases were linear, with the catalytic construct attached to the 5’-end of the oligonucleotide recognition motif [22,23,24]. However, the next generations of ss-aRNase had catalytic constructs incorporated in the middle of recognising oligonucleotide in order to improve catalytic ability and provide an opportunity for catalytic turnover [41]. Presumably, the reduced affinity of the aRNase to the target after each cleavage event facilitates the release of ss-aRNase from the hybridised complex. Such hypotheses turned out to be rather successful and became widely used for the creation of several types of ss-aRNases incorporating different catalytic moieties, some of them demonstrating catalytic turnover [30,37,42].

## 2. Synthetic Approaches Applied for the Generation of Site-Selective Artificial Ribonucleases 

At least three different strategies have been employed for the synthesis of ss-aRNase, which are conjugates of an oligonucleotide recognition motif and some functional groups catalysing RNA cleavage. Historically, the first method applied for the synthesis of ss-aRNase was a fragment conjugation in solution, when the individual structural components (i.e., an oligonucleotide and a catalytic moiety), which were separately synthesised, deprotected and isolated, were then allowed to react with each other in the presence of respective condensing or activation reagents [43,44,45,46]. Nowadays, this approach has been successfully applied for the synthesis of peptidyl-oligonucleotide conjugates of various design [34,36,42,47,48,49,50]. Another version of fragment conjugation, which was widely applied for the synthesis of various ss-aRNases, was also based on the post-synthetic coupling between the key players, when one of the reacting components (usually oligonucleotide) was still bound to the solid support, while the second component (usually catalytic moiety) was in solution [26,30,31,32,37,38,40,51,52,53]. The third major approach was solid-phase synthesis based on the sequential assembly of the oligonucleotide and peptide (or any other RNA cleaving groups) on a single solid support during standard synthesis to generate a complete conjugate structure [33,54,55,56]. Each of these approaches has its own advantages and limitations, which will be discussed below. 

### 2.1. Fragment Conjugation in Solution: Application to Peptidyl-Oligonucleotide Conjugate Synthesis

The synthesis of “single” [34,35,36,50], “hairpin” [29,30,44], “dual” [47] and “bulge-inducing” [42] peptidyl-oligonucleotide conjugates (POCs) was carried out using fragment conjugation in solution. The synthetic peptide was attached to either one (in the case of “singe”, “hairpin” or “bulge-inducing” POCs) or two oligonucleotide recognition motifs (in the case of “dual” conjugates) in DMSO, which often required the use of a DMSO-soluble cetyltrimethylammonium salt of the appropriate oligonucleotide(s). 

In the case of “single” conjugates [28], the formation of the phosphoramidate bond between the 5′-terminal phosphate of the oligonucleotide and the peptide *N*-terminal was usually achieved using the established method of Zarytova et al. [57] with appropriate adjustments due to the presence of a free *C*-terminal carboxylic acid. This method required the use of activating agents (i.e., 2,2′ dipyridyl disulphide, triphenylphosphine and 4-(dimethylamino)pyridine). To prevent peptide self-condensation, the phosphate group of the oligonucleotide was first pre-activated in anhydrous DMSO, and the activated oligonucleotide was isolated by precipitation in diethyl ether prior to the addition of the peptide directly to the activated complex. 

In the case of “hairpin” [29,30,44] and “bulge-inducing” POCs [36], the peptide was attached via its C-termini to the aminohexyl linker located either at the 5′-terminal phosphate (“hairpin” POCs), or at the C8 position of adenosine residue (Type 1 “bulge-inducing” POCs), or at the anomeric C1′ carbon, either in α- or in β-configuration of an abasic sugar residue (Type 2 “bulge-inducing” POCs) located in the middle of the RNA recognition motif. In all these cases, 4-dimethylaminopyridine (DMAP) and *N*,*N*’-dicyclohexylcarbodiimide (DCC) were used as activating agents to promote the amide coupling reaction.

Alternatively, thiol (disulphide protected) modified oligonucleotide was used for the synthesis of some “single” and “dual” POCs [41]. In such cases, the 5′-thiol-modified oligonucleotide, usually supplied as a protected disulphide, was first reduced by tris(2-carboxyethyl)phosphine (TCEP) in phosphate-buffered saline [58]. Then, a maleimide-modified catalytic peptide (either Mal-[LR]_4_G or Mal-[LRLRG]_2_) dissolved in DMSO was added to the oligonucleotide solution for condensation. The synthesis of “dual” conjugates, which consisted of two oligonucleotide motifs connected by a catalytic peptide, required a more complex synthetic scheme. The conjugation of two separate oligonucleotide recognition motifs to the catalytic peptide was carried out in two consequent stages: first via coupling of the first oligonucleotide at the N-terminus, and then via attachment of the second oligonucleotide at the C-terminus of the same peptide. This could be achieved by implementing two different methods which normally require different types of 3′- and 5′-terminal oligonucleotide modifications. Method 1 is based on the formation of a phosphoramidate bond between the 5′-terminal phosphate group of the first oligonucleotide and the peptide *N*-terminal amine [57]. This is followed by conjugation of the second recognition motif via aminohexyl linker located at the 3′-terminus of the second oligonucleotide to the peptide *C*-terminal modification, which can be carried out in 2-(*N*-morpholino)ethanesulphonic acid buffer (pH 6) in the presence of the activating agents water-soluble 1-Ethyl-3-(3-dimethylaminopropyl)carbodiimide (EDC) and *N*-Hydroxy succinimide (NHS). Method 2 utilises a thiol-maleimide ‘click’ reaction between a 5′-thiol modified oligonucleotide (as the first recognition component) and a *N*-Maleoyl-β-alanine residue at the peptide *N*-terminal [59], which can be carried out in phosphate buffered saline aqueous solutions [59,60]. The reaction between the 5′-thiol and maleimide group is spontaneous [59], and the product yields for “single” conjugates were reported to be high (95–100%). The conjugation of the second recognition motif can then be achieved using the same approach as described above for Method 1. 

The main advantage of fragment conjugation in solution is that it offers some freedom in the selection of oligonucleotide length, modification patterns, gapmer or mixmer oligonucleotide organisation, as well as the opportunity to incorporate any type of non-nucleotide inserts, modified nucleobases or RNA cleaving constructs. The only requirement here is the appropriate choice of conjugation conditions and reagents. However, this approach requires multiple and laborious purification stages and often suffers from poor coupling yields. 

### 2.2. Fragment Conjugation on the Solid Support 

The fragment conjugation on the solid support involves the post-synthetic coupling of two oligomeric components, when the first oligomer remains to be linked to the solid support, while the second oligomer reacts in solution. The generated ss-aRNase undergoes deprotection, followed by cleavage from the support, and final purification. This method is considered an ideal method for the wide-scale preparation of various conjugates due to the less laborious purification steps compared to fragment conjugation in solution. 

Several excellent examples of such efficient synthesis of ss-aRNases by fragment conjugation on the solid support were given previously in the series of publications by the groups of Gobel and Stromberg [26,30,31,32,37,39,40,52]. For example, this method was successfully used for the attachment of the RNA cleaving tris(2-aminobenzimidazoles) to DNA oligonucleotides via either disulphide or amide bonds. To avoid the aggregation of negatively charged oligonucleotides with positively charged benzimidazoles, the conjugation methods employed protected oligonucleotides still bound to the solid support. In the first method, a trityl-protected thiohexyl linker was attached to the 5′-terminus as a phosphoramidite building block. After the removal of a trityl protecting group, the resin was treated with the cleaving construct, leading to the formation of a disulphide bridge, which was shown to be sufficiently stable to survive the subsequent deprotection steps. An alternative approach involved the incorporation of an aminohexyl linker protected by a monomethoxytrityl group at the 5′-rerminus of the oligonucleotide, which was deprotected and coupled with the carboxylic acid of the cleaver in the presence of *N*,*N*′-diisopropylcarbodiimide (DIC) and hydroxybenzotriazole (HOBt). This led to the formation of an amide bond, which was sufficiently resistant to chemical degradation during the subsequent deprotection stages [26]. 

A similar strategy was used for the incorporation of the tris(2-aminobenzimidazole) cleaving construct into the fully protected PNA-oligomer, when the latter was still attached to the solid support. To achieve that, the Fmoc-protected 6-aminohexanoic acid was conjugated to the PNA chain at the terminal amino group to generate an aminohexyl linker. After removal of the Fmoc protecting group from the aliphatic amine, it was allowed to react with the carboxylic functional group of tris(2-aminobenzimidazole) derivatives via amide-coupling reactions in the presence of DIC and HOBt activating agents, thus leading to 100% yield, as no unconjugated PNA was detected in the reaction products [30,31]. 

The neocuproine-based ss-aRNases were also synthesised by fragment conjugation on the solid support [37,38,52,61] by utilising PNA sequences with an internally placed diaminopropionic acid (Dap) unit in a position facing the bulge in the target upon hybridisation, which also serves as an attachment point for the catalytic group via the side chain amino group. The PNA–Dap–PNA oligonucleotide analogues were synthesised on the solid support using Fmoc chemistry, followed by deprotection of the Dap unit to generate free amine, which then was exposed to the coupling reaction with phenyloxy carbonyl-5-amino-2,9-dimethylphenanthroline, either directly or after extension of the Dap unit with a glycine moiety. This allowed a series of 2,9-dimethylphenanthroline (neocuproine) conjugates to be generated [52,61,62]. 

Recently, a similar approach was successfully implemented to produce structurally different neocuproine-containing ss-aRNases bearing an additional oligoether group to enhance its cleavage potential [38]. This approach was based on the post-conjugation of polyethers 2-(2-(2-(benzoyloxy)ethoxy)ethoxy)acetic acid (PE) and 5-phenoxycarbonylamino-2,9-dimethyl-1,10- phenanthroline to PNA still bound to the solid support. The terminal Fmoc was cleaved off the respective PNA, and PE pre-activated with 2-(1*H*-Benzotriazol-1-yl)-1,1,3,3-tetramethyluronium Hexafluoro-phosphate (HBTU) and HOBt was conjugated to the deprotected amine group. Once the terminal polyether arm was added to PNA, the N^β^-methyltrityl protecting group was removed. One HN-Lys(^ε^N-Mtt)OH was then coupled to the PNA, and a second unit of PE was attached to the ^ε^N of the lysine residue. After removal of the ^ε^N-methyltrityl protection of the Lys, the support was subjected to reaction with 5-phenoxycarbonylamino-2,9-dimethyl-1,10-phenanthroline (in the presence of 4-methylmorpholine (NMM) and *N*-Methyl-2-pyrrolidone (NMP) as condensing agents). The resultant PNA conjugate was deprotected and cleaved from the solid support. A very similar synthetic procedure was applied to prepare PNA conjugates with neocuproin and *H*-His(Trt)-OH. In this case, after conjugation with neocuproin, the terminal Fmoc protection was cleaved off and the poly-His peptide was synthesised on the terminal part of the PNA.

The 2′-OMe oligoribonucleotide conjugates bearing two azacrown ligands attached via a phosphodiester linkage to a single non-nucleosidic building block were assembled using conventional phosphoramidite chemistry adapted to standard RNA coupling [63]. To achieve that, three different non-nucleosidic branching units were prepared and converted to 4,4′-dimethoxytritylated 2-cyanoethyl *N*,*N*-diisopropylphosphoramidite building blocks. The two hydroxyl groups in each building block, which were expected to be engaged in successive conjugation with the azacrown ligands, were protected as levulinic acid esters. Once the 2′-OMe oligoribonucleotide chains were assembled, the levulinoyl protection groups were manually removed, and deprotected resin-bound oligonucleotides were reset to the DNA synthesiser where the azacrown phosphoramidite reagent was then coupled using two consecutive standard couplings. Fully protected resin-bound 2′-OMe oligoribonucleotide conjugates were released from the support and deprotected by concentrated ammonia.

An interesting design of ss-aRNases was proposed in [64]. This ss-aRNAases were composed of a PEG–PNA–PEG domain conjugated to cleaving groups (either to short peptide HGG⋅Cu or to diethylenetriamine (DETA)). In this case, the polyethylene glycol units were introduced at both the C- and N-termini of PNA oligomers, in order to improve the PNA aqueous solubility and to separate the catalytic domain the RNA binding motif. The conjugates were synthesised using standard solid phase peptide synthesis. At the initial step, Rink-amide resin was functionalised with Fmoc–PEG_2_–COOH in the presence of 1-[Bis(dimethylamino)methylene]-1*H*-1,2,3-triazolo[4,5-b]pyridinium 3-oxide hexafluorophosphate (HATU) activating agent, followed by deprotection, when the free amino group of the growing chain was used to synthesise PNA oligomer by incorporating Fmoc–PNA–(Bhoc)–OH building blocks. This was followed by the incorporation of the second Fmoc–PEG_2_–COOH fragment at the N-terminus of PNA to give the binding domain PEG–PNA–PEG of the ss-aRNases A and B. The synthesis of ss-aRNase A was finalised by manual coupling with Fmoc–His(Trt)–OH and Fmoc–Gly–OH amino acids to produce the HGG domain at the N terminus of PNA chain. In the case of ss-aRNase B, following deprotection of the PEG linker, the Fmoc–Lys–(MTT)–OH amino acid was first incorporated to provide a suitable attachment point for conjugation with the catalytic domain. After deprotection of the ε-amino group of the incorporated lysine, the resin was subjected to treatment with (Boc)_2_–DETA–Succ–OH from solution to generate the desired conjugate.

### 2.3. Solid-Phase Synthesis 

In the solid phase synthesis, a peptidyl oligonucleotide conjugate (POC) is generated through the sequential assembly of a peptide and oligonucleotide on a solid matrix. A reactive but masked functional group is present at the conjugation site of the peptide. The points of conjugation are usually the termini or side chains of the oligonucleotides and peptides, respectively. The *peptide-oligonucleotide* assembly can follow one of the two patterns, either “*oligo-first-peptide-next*” or “*peptide-first-oligo-next*”. Predominantly, peptides are synthesised first using a Boc or Fmoc synthetic strategy, followed by oligonucleotide conjugation via the phosphoramidite method [54].

The lack of mutual compatibilities in the reaction conditions necessary for the synthesis of oligomers witnessed in the solid-phase method is one of the major bottlenecks of this method because acid-labile oligonucleotides and peptide chains are not always stable when subjected to the relatively harsh reaction conditions that are essentially used for the synthesis of either peptide or oligonucleotide fragments, respectively. Therefore, finding the correct protecting group compatible with both oligonucleotide and peptide moieties is the main challenge of solid-phase synthesis. Besides, only amino acids with no reactive side chains like alanine and leucine or those with easily removable side chain protection groups can be conjugated with oligonucleotides via the solid phase method [54,65]. At present, no POC introduced as an ss-aRNase has been prepared by solid phase methods.

On the contrary, the solid phase method was shown to be very efficient in the synthesis of imidazole-containing oligonucleotide conjugates. In this series, ss-aRNases have been prepared using a two-step procedure. In the first step, the 17-mer oligonucleotide recognition motif, which was synthesised using standard solid-phase phosphoramidite chemistry, was then extended (at the end of the synthesis) with methoxyoxalamido (MOX) modifiers of a different type. In the second step, the prepared oligonucleotide–MOX precursors were then functionalised with 2 M histamine solution in dimethylformamide, followed by deprotection, to yield the oligonucleotide conjugates, bearing between 2 and 32 histamine residues at the 5′-terminus of the recognition oligonucleotide. The distance between oligonucleotide and imidazole residues of the cleaving part can be varied by altering the structures of the anchor and the linker groups [33,55,56].

## 3. In Vitro Characteristics of Artificial Ribonucleases

### 3.1. Chemical Moieties Used for Creating aRNases and the Corresponding Mechanism of Cleavage

Artificial RNases cleave RNA phosphodiester bonds by catalysing an intramolecular transetherification reaction caused by the nucleophilic attack of 2’-oxygen on the adjacent phosphorus centre. This reaction has been thoroughly analysed elsewhere [66,67] and a comprehensive analysis of aspects concerning aRNases was reported by Lönnberg’s group [21,68], so will not be considered here. However, it is important to emphasise that the RNA cleavage reaction might be facilitated via one of four independent methods (or their appropriate combination): (i) by accelerating deprotonation of the attacking nucleophile (2’-OH), (ii) by protonation of the departing nucleophile (5’-*O*-), (iii) by protonation of the non-bridging phosphoryl oxygen, and (iv) by promoting the ‘in-line’ geometry necessary for the 2’-OH attack of the bridging phosphorus atom [66]. Artificial RNases exploit one or several of these catalytic mechanisms in a co-operative manner depending on the type of the catalytic groups involved.

#### 3.1.1. Acridines and Azacrowns

Various types of acridine groups can be used for site-selective RNA scission as a part of the DNA–acridine conjugate, which normally act in cooperation with free lanthanide (III) ions or various divalent ions (e.g., Zn(II) and Mn(II)) (Figure 1, Table 1) [69,70]. The intercalation of acridine in the RNA–DNA backbone of the hybridised complexes induces conformational changes, presumably via promoting an ‘in-line’ geometry and may then facilitate the cleavage of adjacent phosphodiester linkages in the presence of metal ions. At the same time, acridine acts as an acid catalyst, thereby activating at least two mechanisms of catalysis. The acid catalysis requires certain substituents. However, acridine-bearing variants showed a relatively low cleavage efficiency and require either lanthanide (III) [70] or Zn(II) [69] ions for catalysis. This limits the opportunity for acridine–DNA conjugates to be used in cell culture or in vivo studies.

Ss-aRNases based on 2’-*O*-Me oligoribonucleotides, used as a recognition motif, and two 3-(3-hydroxypropyl)-1,5,9-triazacyclododecane (azacrown) ligands, attached as a catalytic moiety via a phosphodiester linkage to a single non-nucleosidic building block, were investigated by Lönnberg’s group [63] and appeared to be more efficient than acridine-based ones. These conjugates were designed against chimeric oligoribonucleotides, consisting of 9-2’-*O*-Me and 11-2′-Hydroxy ribonucleotides. Several variants of attachment were studied, including the incorporation of one or two azacrown ligands, which were located either close to the 5’-end of targeting oligonucleotide or close to its central part. The best variants showed cleavage efficiency with a half-life less than 10 h that is comparable with the best variants of the other types of Cu^2+^ and Zn^2+^ ion-dependent conjugates. As expected, all of the di(azacrown) appeared to be better catalysts than their mono(azacrown) counterparts. Importantly, the catalytic turnover was demonstrated for two conjugates.

#### 3.1.2. Trisbenzimidazole

Tris(2-aminobenzimidazole) was found to be one of the most effective metal-ion free cleavers of phosphodiester linkages investigated as a domain of aRNases. The screening of several derivatives of 2-aminopyridine and tris(benzimidazoles) in the pioneering work allowed the most promising structural variants to be selected [53]. Tris(2-aminobenzimidazole) appeared to be the most successful cleaver, and was used to create ss-aRNases. Initially, these aRNases were less effective than metal-ion dependent ones, showing a relatively long half-life of 12–17 h for tris(2-aminobenzimidazole) as compared to only 2 h for lanthanide(III)-ion dependent aRNases [26]. However, their efficiency was eventually improved to achieve a half-life of 3.5 h. The main factor that allowed this improvement was the nature and structure of the recognising oligonucleotide. By switching from DNA to DNA-PNA mixmers, it was possible to considerably decrease the half-life of the RNA target upon treatment with these ss-aRNases [32].

#### 3.1.3. Peptide [(LeuArg)_n_Gly]_m_

**Figure 2:**
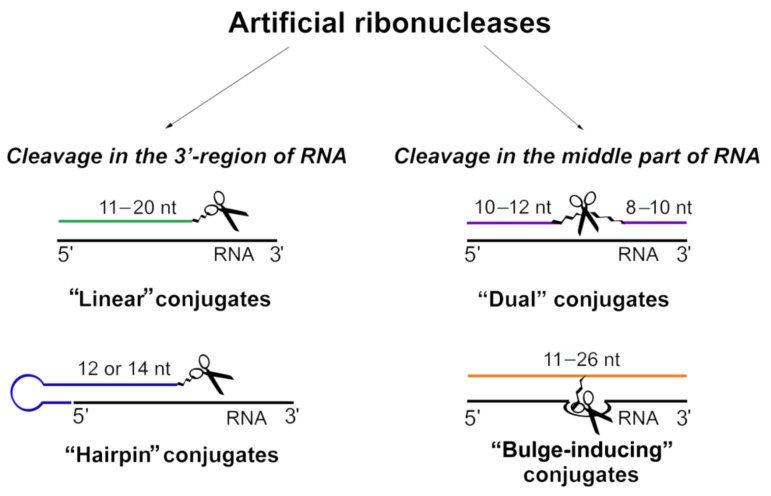
Schematic representation of duplexes of ss-aRNases of various design with RNA targets. Numbers show the length of RNA recognition motif complementary to target sequence within RNA. Scissors refer to a catalytic domain of ss-aRNase.

Peptidyl-oligonucleotide conjugates (POCs) represent another large class of ss-aRNases, which are based on incorporation of the catalytic peptide into the oligonucleotide recognition motif(s) at different positions using a broad variety of attachment methods [34,35,36,42,47,50]. Among others, peptides with alternating leucine and arginine residues [(LeuArg)_2_Gly]_2_ and [(LeuArg)_4_Gly] proved to be the most efficient cleavers as part of the ss-aRNase subject to the certain rules of conjugate design. For example, “bulge-inducing” POCs (see Section 2.2 and Figure 2) require long flexible linkers such as an aminohexyl moiety. Moreover, the conjugation point seems to be crucial for ribonuclease activity. Indeed, the conjugates with an aminohexyl linker attached at the C1′ position of the anomeric sugar in either the α- or β-configuration appeared to be effective cleavers (with half-life for target RNA of 8 h). In contrast, the attachment of the same peptide to the aromatic ring (e.g., C8-position of adenosine) showed zero cleavage activity [42].

The aminohexyl linker seems to provide a sufficient flexibility to catalytic groups, thus facilitating phosphodiester bond cleavage. Another way to enhance the overall flexibility of a catalytic peptide could be the insertion of an additional glycine residue, which serves as a flexible joint between two Leu-Arg-Leu-Arg blocks. Thus, the probability of achieving an ‘in-line attack’ conformation can be increased to promote catalytic activity. Indeed, the conjugates incorporating the peptide [(LeuArg)_2_Gly]_2_ generally exhibited increased [42,47] or at least equal [34] activity to those based on the peptide [(LeuArg)_4_Gly].

Replacement of the *C*-terminal carboxylic acid group with a carboxamide group resulted in a surprisingly strong enhancement of RNA cleavage, thus leading to the assumption that the carboxamide group may contribute to the catalysis of phosphodiester transesterification reactions.

The length of the catalytic peptide (and/or the number of the catalytically important groups) seems to strongly correlate with the cleavage activity of POCs. Indeed, linear ss-aRNase incorporating a very short peptide [LeuArg]_2_Gly demonstrated near-zero cleavage activity against target tRNA^Phe^, while the ss-aRNase incorporating a 2-fold longer peptide [(LeuArg)_2_Gly]_2_ cleaved up to 100% of target tRNA^Phe^ within 4 h [34]. This correlates well with the above data seen for di(azacrown) compared to their mono(azacrown) counterparts. One possible explanation could be the assumption that efficient cleavage requires the simultaneous activation of two catalytic mechanisms, for example, deprotonation of the attacking nucleophile (2’-OH) and protonation of the non-bridging phosphoryl oxygen 5’-*O*-, which could be possible only if several catalytic groups are present in the same cleaving domain.

### 3.2. RNA-Recognition Domains of ss-aRNases: Structure and Modification

The essential component of ss-aRNAses, which provides specific binding with a target, is the oligonucleotide-based RNA recognition motif (ON). According to the structure of ON, established ss-aRNAses can be divided into four major groups: (1) “linear”, (2) “dual”, (3) “bulge-inducing” and (4) “hairpin” (Figure 1).

Most of the ss-aRNases belong to the group with a “linear” RNA recognition motif. In these conjugates, the oligonucleotide of 11–20 nts in length forms a perfect complementary duplex with the target RNA sequence (Figure 1 and Figure 2). As a result, the single-stranded region of RNA target remains accessible for cleavage by the catalytic moiety of ss-aRNAses. Developed linear conjugates, in general, contain PNA, LNA or 2′-OMe oligonucleotide analogues as an RNA recognition motif, but can also contain non-modified DNA oligonucleotides. The catalytic domain of these ss-aRNases is presented by tris(2-aminobenzimidazole)s, Zn-dependent azacrowns,, diethylenetriamine, imidazole and peptides [(ArgLeu)_2_Gly]_2_, [His(Gly)_2_] (Table 1) [26,33,34,63,64]. As RNA targets for “linear” conjugates, various short synthetic RNAs, model RNA transcripts (tRNA^Phe^) or biologically relevant RNAs, for instance, miRNAs, were used.

In “dual” ss-aRNases, designed to target yeast tRNA^Phe^ or oncogenic miRNAs, the RNA recognition motif is split into two oligonucleotide shoulders complementary to the 5′- and 3′-extremeties of RNA targets, while the catalytic group is placed between them opposite a short (3–5 nts) single-stranded gap, which is formed upon binding with the RNA target (Figure 1 and Figure 2). Depending on the RNA substrate, the length of each oligonucleotide shoulder varies from 8 to 12 nts and consists of either non-modified or 2′aminoadenines-modified oligonucleotides (Table 1) [47,48].

Another type of ss-aRNAses is “bulge-inducing” conjugates (Figure 2) which include conjugates of tris(2-aminobenzimidazole) and PNA oligonucleotides as well as peptide [(ArgLeu)_2_Gly]_2_ and non-modified DNA analogues targeted to synthetic RNA substrates or tRNA^Phe^, respectively (Table 1) [31,42]. The RNA recognition motif of these ss-aRNases is presented by “linear” oligonucleotides complementary to RNA targets in such a way to form 3–5 nt bulge loops in the target upon binding with the conjugate (Figure 2). The catalytic group of “bulge-inducing” ss-RNAses is located opposite the forming bulge loop, and the cleavage of RNA usually occurs at one or several bonds within the bulge loop (Figure 2).

The fourth group of developed ss-aRNases contains a “hairpin” oligonucleotide as an RNA recognition domain (Figure 1). The structure of such oligonucleotides includes a purine-rich hairpin with 6 or 9 bp stem and a 12–16-mer single stranded fragment complementary to the 5′-end of the target that mediates enhanced binding with RNA substrates (Figure 2) [35,36,49,50]. A hairpin adjacent to the RNA binding sequence increases the stability of the duplex by stacking interactions [71] and significantly enhances the nuclease resistance of ss-aRNases [36,50]. The most active “hairpin” ss-aRNases contain natural oligodeoxyribonucleotides or 2′-OMe-modified oligoribomicleotides [49]. The cleavage of RNA with “hairpin” ss-aRNases is achieved by catalytic peptides and takes place in a 6–8-mer free single stranded region at the 3′-end of miRNA-targets (Table 1 and Figure 2).

#### 3.2.1. Principles of Short RNA Target Cleavage

Today, the majority of developed ss-aRNases are targeted mainly to short synthetic model RNAs of 15–30 nts in length. However, there is a number of ss-aRNases aimed at degrading biologically relevant short RNAs, namely, microRNAs. Extremely small length (21 nts) of such RNAs significantly complicated the design of ss-aRNases because such ss-aRNAses should leave a single-stranded region of RNA substrate accessible for cleavage upon binding with the target. At the same time, it should maintain the balance between effective duplex formation and the dissociation of ss-aRNase from a complex with RNA after cleavage to maintaining the catalytic mode of action.

Nowadays, several research teams have successfully developed ss-aRNases targeted to short RNAs. For instance, the group of Lönnberg designed Zn-dependent ribonucleases based on 3-(3-hydroxypropyl)-1,5,9-triazacyclododecane (azacrown) and “linear” 18-mer 2′-OMe-oligonucleotides. Such conjugates targeted to 36-mer synthetic chimeric 2′-OMe-RNA substrate promote 90% target cleavage within 120 h [63].

M. Göbel et al. designed “linear” ss-aRNAses consisting of tris(2-aminobenzimidazole) and 15-mer DNA, PNA or DNA-LNA mixmer oligonucleotides targeted to 22–29-mer fragments of 3′-UTR PIM1 mRNA [26,30,32,72]. The half-lives (τ½) of the cleavage of DNA- or PNA-ss-aRNases vary from 11.2 to 16.5 h [26,30,32,72]. In turn, conjugates of DNA-LNA mixmers exhibit significantly faster cleavage of RNA with τ½ 3.5 h [26,30,32,72]. It should also be noted that another conjugate developed by this group, the “bulge-inducing” PNAzyme targeted to 15-mer synthetic RNA, is able to degrade up to 70% of the 5-fold RNA excess within 84 h in a catalytic manner, which shows a significant achievement for metal-independent PNA-based ss-aRNAses [31].

In the group headed by R. Strömberg, Cu- and Zn-dependent ss-aRNAses are developed based on dimethylphenantroline (neocuproine) and “bulge-inducing” PNA oligonucleotides. Such conjugates target a short 15-mer model RNA, the sequence of which corresponds to the junction of bcr/abl mRNA [52]. Irrespective of the linker structure and the size of the bulge forming, Zn-dependent aRNases are characterised by τ½ 11–15 h [52]. The most effective Zn-dependent aRNases exhibit 50% cleavage of target within 7-8 h [39] and are able to degrade the 4-fold excess of substrate in a catalytic mode [52]. The replacement of Zn^2+^ ions with Cu^+^ leads to a manifold increase in the cleavage rate by neocuproin-based conjugates. Such PNAzymes forming a 4-nt bulge-loop are characterised by a τ½ equal to 1.5–3 h [38] whereas the most effective Cu-dependent aRNase demonstrates a much faster cleavage kinetic, degrading 50% of the 100-fold excess of the substrate in 0.5 h [37].

A vast amount of data provides evidence that short RNAs such as miRNAs, piwi-associated-RNAs and small nucleolar RNAs stimulate tumorigenesis, suggesting these molecules as perspective therapeutic targets for oligonucleotides-based inhibitors including ss-aRNases. Already designed short RNA-targeted conjugates mostly address the oncogenic miRNAs and are divided into three groups according to the structure of the oligonucleotide domain: (1) “linear”, (2) “dual” and (3) “hairpin” (Figure 1 and Figure 2).

Investigation of the ribonuclease activity of “linear” aRNases shows its relatively high efficiency. In particular, miR-21-targeted conjugates of 16-mer DNA oligonucleotide and a peptide [(ArgLeu)_2_Gly]_2_ developed by our group quantitatively cleave the miRNA target with τ_½_ 15.1 h (Table 1) [35]. Created by Gaglione and co-authors, conjugates of a 14-mer PNA oligonucleotide and tripeptide [His(Gly)_2_] or DETA provide the cleavage of miR-1323 by 47.5% and 90% within 24 h, respectively (Table 1) [64]. miR-20a-targeted aRNases based on a 15-mer PNA oligonucleotide and tris(2-aminobenzimidazole), designed by Danneberg and co-authors, achieves 85% target cleavage within 60 h (Table 1) [30].

The second group of miRNA-targeted ss-aRNases are “dual” conjugates targeted to oncogenic miR-17, miR-21, miR-18a and miR-155 [48]. These conjugates possess high affinity to the targets due to 2′-aminoadenine modifications that increase duplex stability. The maximal level of miRNA cleavage observed for “dual” conjugates amounts to 57% in 48 h (Table 1).

The third type of developed miRNA-targeted ss-aRNases consists of catalytic peptide and hairpin oligodeoxyribonucleotides in which a 12-mer fragment is complementary to miR-17 or miR-21 and a peptide Gly(ArgLeu)_4_ is attached to oligonucleotide via a thymidylate bridge [36]. Such compounds provide 50% miRNA cleavage in 72 h (Table 1) [36]. The highest ribonuclease activity among the developed hairpin miRNA-targeted ss-aRNases possess conjugates of peptide [(ArgLeu)_2_Gly]_2_ and DNA oligonucleotides with a 14-mer fragment, complementary to miR-21 (Table 1). Such design maintains extremely high ribonuclease activity of compounds: a 2–10-fold excess of miRNA was cleaved by up to 87% within 72 h [35].

#### 3.2.2. Principles of Long RNA Target Cleavage

Speaking of ss-aRNases, we always remember that they can be multifunctional instruments, making subtle manipulation with targeted RNA possible; however, their main objective is to cleave selected RNA in vivo, influencing various biological processes. Thus, all studies devoted to ss-aRNases imply the targeting of biologically relevant RNAs in the long-term. The cleavage of long mRNA transcripts causes additional difficulties because the secondary and tertiary RNA structures would prevent the binding of ss-aRNases with it. Since the review of Lonnberg [21], when there were only a few papers, where metal-dependent ss-aRNases cleaved biologically relevant RNA transcripts of human c-raf-1, telomerase and apolipoprotein E gene [46,73,74], the situation has not changed dramatically. The ss-aRNase based on tris(2-aminobenzimidazole) is one of the few examples showed the ability to efficiently cleave long structured biologically relevant RNAs representing 155, 412 and 430-mer RNA transcripts derived from the 3′-UTR of the PIM1 mRNA [32].

Studies of tRNA^Lys^ [75] and tRNA^Phe^ [34,42,47] cleavage with POCs showed that, in contrast to other types of ss-aRNases such as based conjugates of imidazol [33] or tris(2-aminobenzimidazole) [32], POCs cleave selected RNA not only at the target site but also outside it. The cleaved sites are pulled together due to the tertiary RNA structure and are therefore accessible for catalytic peptides. This regularity was only observed for POCs. Most likely, the extended peptide is prone to cleaving distant regions, which could be an advantage for long RNA inactivation. Nevertheless, a similar capacity of non-target distant region cleavage for ss-aRNases contained imidazol, tris(2-aminobenzimidazole) or other catalytic groups are worthy of notice.

### 3.3. Specificity of Cleavage

#### 3.3.1. Site-Selectivity

On par with efficiency, site-selectivity is one of the main characteristics of ss-aRNases. It implicates the extent of target RNA cleavage at preferential positions or nucleotide bases. In natural enzymes, the amino-acid composition of active sites determines the sequence-specificity of catalysis. In general, the majority of natural enzymes possess pyrimidine-A (Pyr-A) specificity. Usually, the His residues are responsible for cleavage after pyrimidine bases, as in the case of RNase A [76]. However, in some enzymes, Asp, Lys and Asn may also drive the cleavage at Pyr-A sites [77]. Several ribonucleases, including members of the RNase T1 family, possess G-X specificity of RNA cleavage [78]. Most likely, the presence of arginine in the active site of RNase T1 promotes the formation of arginine-fork structures in the duplex with RNA. In such cases, the networks of hydrogen and van der Waals bonds between the guanidinium group of Arg and the oxygen and nitrogen of guanine bases are evolving, followed by cleavage at corresponding G-X bonds [79].

Most often, ss-aRNases mimic the catalytic sites of natural enzymes, although the composition and design of conjugates may affect the specificity and efficiency of cleavage. For instance, conjugates containing imidazole residues as catalytic domains imitate the active site of RNase A. Such ss-aRNases, as shown in [33,80], exhibited Pyr-A specificity during tRNA cleavage. In turn, the specificity of conjugates comprised of more sophisticated imidazole-based catalytic domains, such as tris(2-aminobenzimidazole), was not limited by pyrimidine-A cleavage specificity, but also include degradation at purine-X motifs [26,30,31,32,72]. Established in our group, conjugates of the peptide [(ArgLeu)_2_Gly]_2_ attached to the hairpin oligonucleotide via an aminohexyl linker cleaved miR-21 exclusively at G-X bonds, representing mimics of RNase T1 [35]. However, moderate alterations in the peptide composition and changes of the linker type led to a switch of specificity from G-X to Pyr-A [36]. In the study of a new series of conjugates, we discovered that ”dual” ss-aRNases possess Pyr-A specificity, although the same peptide as in the case of hairpin aa-aRNases was used [48]. Since miR-21 was not initially subjected to cleavage by “dual” conjugates, we assumed that a fissionable single-stranded gap of natural miRNA does not include pyrimidine-A sites. The alterations of gap miRNA sequences to AUACA resulted in the increase in cleavage efficiency from 0 to 18% within 48 h [48], confirming the Pyr-A preference of “dual” miRNA-targeted conjugates.

The data of Staroseletz et al. demonstrated that the distance from the attachment point of the catalytic group to the cleavage site may also influence the specificity. In particular, tRNA-targeted “bulge-inducing” and “dual” ss-aRNases exhibit wider specificity when the target site is located opposite the catalytic group, promoting cleavage at Pyr-A motifs as well as at A-C bonds [42,47], whereas the degradation of tRNA in the distant area by such conjugates occurs at easily degraded Pyr-A sites or at G-X bonds, as exceptionally observed for “dual” conjugates [42,47].

The thorough study of cleavage structure-specificity was conducted for Zn^2+^ and Cu^+^-dependent 2,9-dimethylphenanthroline “bulge-inducing” PNAzymes [39,40]. Using the synthetic fragment of bcr/abl derived mRNA, the authors investigated the influence of size and sequence of bulge-loop induced in target RNA on the efficiency and specificity of cleavage. The impact of each nucleotide substitution in 3 or 4 nt bulge-loops started from a tri- or tetra-A stretch was evaluated, taking into account that the complex contains a wobble base pair closing the bulge-loop [39,40]. It was found that the highest site specificity in the systems with a 4 nt-bulge-loop was observed for the -ACAA- sequence, resulting in 70% cleavage at a single A-A bond. In turn, the maximum site specificity in the 3 nt-bulge-loop was observed for -AUA-, -GUA- and -UUA-sequences, resulting in 80% at the U-A bond. For both types of PNAzymes, the contraction of bulge size and replacement of adenosine in the position next to wobble pair dramatically decreased the rate of cleavage. Moreover, substitutions of purines to pyrimidines in different positions of the bulge-loop sequence significantly changed the ribonuclease activity of such metal-dependent ss-aRNases [39,40]. Most likely, stacking between two nucleotides closest to the wobble pair, their interactions with the catalytic group and stacking with the wobble pair influence the rate of cleavage by PNAzymes.

It should also be noted, that ss-aRNase performance depends greatly on the target sequence. As shown for tris(2-aminobenzimidazole)-based ss-aRNases, τ½ of cleavage can be improved from 10 to 3 h by changing only the target sequence while retaining the same catalytic group and conjugate design [32]. A similar tendency was also observed for “dual” conjugates, where differences in target miRNA sequence may be a reason for cleavage efficiency scattering up to 30% [48].

To sum up, three main parameters define the specificity of target RNA cleavage by ss-aRNases, including: (1) the structures of catalytic group and a linker; (2) the general design of conjugates (“hairpin”, “dual”, “bulge-inducing” or “linear”); and (3) the target RNA sequence.

#### 3.3.2. Non-Complementary Substrates

Another important issue concerning ss-aRNases is the cleavage of non-complementary substrates. Although ss-aRNases are intended to cleave target RNA only upon forming a complementary complex, the opportunity for non-specific cleavage cannot be ruled out. Obviously, the cleavage of non-complementary targets may cause undesirable side effects, so it is therefore necessary to try to avoid them. Sometimes it is possible to achieve this goal [32,34]; however, some ss-aRNases exhibit the ability to degrade non-complementary RNAs [42]. The cleavage of non-complementary or partially complementary targets is less efficient than the cleavage of fully complementary targets. Moreover, it is pretty naive to expect the catalytic moiety to remain inactive against all RNAs in view of the unspecific and imperfect complementary interactions. It is particularly true with regard to large and positively charged scissors as a peptide [(ArgLeu)_2_Gly]_2_. Bearing this in mind, the main question is whether the utilised ss-aRNase cleaves off-target RNAs with an efficiency comparable with that of target RNA cleavage. To date, the only work to have studied this issue argues that the effect of non-specific cleavage can be neglected [35]. The studied miRNA targeted ss-aRNases effectively decreased the level of target miR-21 in lymphosarcoma cells RLS_40_ while the level of several other miRNAs (let7-g, miR-17 and miR-18a) remained unaltered [35].

### 3.4. Chemical Modifications of Oligonucleotide Domain: Influence on ss-aRNase Performance

It is noteworthy that the nature of the recognition domain of an ss-aRNase can play a crucial role in its clinical performance. Favourable properties increasing the therapeutic potential of aRNases are the enhanced resistance of the oligonucleotide component to nucleases, high affinity to the target RNA and increased penetrating ability. These capabilities may be provided by substitution of the DNA scaffold by DNA/LNA, and 2-OMe and PNA-modified oligonucleotides used as the binding domains in the developed conjugates (Table 1).

In contrast to DNA oligonucleotides, which undergo rapid digestion by intracellular nucleases, the special advantages of 2′-OMe, LNA and PNA oligonucleotides are enhanced nuclease resistance and a high affinity for the target RNA. Since even a small change in the conjugate structure may significantly impair its catalytic performance, one of the main issues in the design of ss-aRNases is the influence of chemical modifications on their ribonuclease activity.

The catalytic properties of tris(2-aminobenzimidazole)-based ss-aRNases, which contain different patterns of LNA modifications of oligonucleotide domain, were also investigated [72]. It was shown that the introduction of one LNA modification does not influence the ribonuclease activity of conjugate: τ½ of DNA and LNA-modified ss-aRNase were 14 ± 0.9 h and 13.3 ± 0.8 h, respectively [72]. The addition of two or three LNA units promoted a 2-fold increase in the rate of cleavage, resulting in τ ½ equal to 6.4 ± 0.2 h [72]. The introduction of 6 LNA units leads to the additional enhancement of ribonuclease activity of ss-aRNase, lowering its τ ½ to 3.5 ± 0.4 h [72]. Moreover, depending on the sites of modification, different RNA cleavage patterns were observed. DNA ss-aRNase or conjugates containing 6 LNA substitutions cleaved the RNA substrate at 2 bonds located in the single stranded region of the RNA and at two additional sites located within the duplex with the conjugate. To compare, ss-aRNases with two or three LNA modifications cleaved one bond within the bulge-loop and three bonds out of the duplex [72].

Our studies showed that “hairpin” ss-aRNases containing a fully 2′-OMe-modified RNA recognition domain led to a substantial decrease in ribonuclease activity compared to unmodified analogues: the maximum cleavage efficiency did not exceed 81% in 72 h [49]. In contrast, partial modification, leaving the 3 nts adjacent to the catalytic peptide attachment point unchanged, stimulate miRNA cleavage by the conjugate: the complete degradation of RNA target was reached at 48 h for the DNA analogue and at 24 h for the partially modified ss-aRNase [49].

An investigation by Danneberg et al. demonstrated that the structure of modified oligonucleotides may also influence the specificity of cleavage. It was found that elongation from 10- to 15-mer of PNA oligonucleotides in tris(2-aminobenzimidazole)-based ss-aRNase results in the loss of site-selectivity of cleavage, leading to the cleavage of target RNA, not only at specific linkages but at all available sites in its single-stranded region. Moreover, lengthening of the PNA oligonucleotide contributes to the formation of aggregates leading to the decrease in effective conjugate concentration in solution [30].

Thus, chemical modifications of the RNA recognition domain, indeed, influence the efficiency and specificity of RNA cleavage by ss-aRNases. To enhance the performance of chemically modified ss-aRNAses, the screening for optimal lengths of oligonucleotide parts should be conducted. Moreover, modified analogues may replace not all of the nucleotide backbone, but should be introduced in certain positions alongside the catalytic group attachment point. Such a strategy seems to be promising since it does not impede the catalytic turnover of conjugates as a result of the less robust binding with the RNA target compared to fully modified analogues [32].

## 4. Therapeutic Application of Sequence-Specific aRNases in Cell Cultures and In Vivo

The fruitful work of the last decade has led to the development of highly potent ss-aRNases that are capable of the sequence-specific multi-turnover destruction of target RNA. Developed synthetic enzymes with desired biocatalytic properties are an indispensable tool for in vitro manipulation with RNA. However, a more vital application of ss-aRNases is their use for therapeutic purposes as highly selective inhibitors of disease-relevant RNAs. The multiple catalytic turnover, underlying the functioning of ss-aRNases, offers potential therapeutic opportunities for using a substoichiometric amount of a drug-enzyme in relation to a target RNA, allowing the irreversible elimination of multiple copies of disease-associated target molecules.

Despite the high efficiency and prospects of ss-aRNases application, data on their activity in eukaryotic cells are extremely sparse. At the beginning of the century, a few reports provided evidence of the successful use of ss-aRNases in cell cultures. It was shown that the conjugate of 2′-*O*-(2-methoxyethyl) oligonucleotide with the lanthanide macrocyclic complex Eu(THED)^3+^ leads to a significant decrease in the level of ICAM-1 protein in endothelial cells [81], and the ss-aRNase based on the phosphorothioate oligonucleotide and the imidazole residue effectively suppresses the replication of the human immunodeficiency virus HIV-1 in MT-4 cells due to the sequence-specific cleavage of HIV-1 gag mRNA [82]. These studies demonstrated the superiority of ss-aRNases in comparison with antisense oligonucleotides and the prospects of their use for therapeutic purposes. Since then, new artificial enzymes with improved properties have been developed; however, the field of application of ss-aRNases as therapeutic agents remains unexplored.

The key condition for therapeutic efficiency of ss-aRNases is the preservation of their stability and activity in the biological medium, including temperature, pH and concentrations of various cations. Huge hopes are inspired by ss-aRNases developed in the past fifteen years (Table 1), since scientists have managed to create compounds that can efficiently function in a neutral pH range and independent of the concentration of magnesium ions, which is a significant achievement on the way to the biological use of engineered ss-aRNases. It should be noted that among the ss-aRNases developed today, it is not possible to single out a leader structure. Among all structure types of ss-aRNases, including conjugates of metal complexes, imidazoles or catalytic peptides, there are highly active constructions that cleave the target in a multi-reaction turnover manner within 0.5–5 h [32,33,34,37,38,40,47,49,72]. However, the use of metal-dependent ss-aRNases in vivo can be a problem due to the possibility of competing interactions with other bioavailable metals in the cell, as well as with various protein ligands, which makes them practically uncontrollable. Moreover, the destabilisation and loss of metals from their coordinating ligands raises concerns about their toxicity to humans. Metal-independent ss-aRNases, such as conjugates of imidazoles, cationic amines and peptides, are promising candidates for therapeutic application, as these compounds are less toxic and more stable in intracellular conditions.

**Figure 3:**
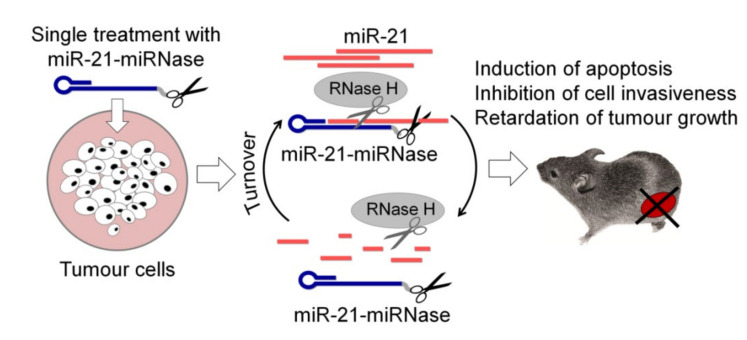
Anti-tumour effect of miR-21-targeted miRNase, achieved due to the synergistic degradation of miRNA-target by jointly miRNase and RNase H in a multi-turnover reaction.

Despite the outstanding achievements in the development of ss-aRNases working in vitro, only conjugates carrying short peptides as a catalyst have been tested as inhibitors of pathogenic RNA in vivo. Our research group has recently designed peptide-based ss-aRNases aimed at inhibiting oncogenic miRNAs in tumour cells, which were termed miRNases [35,49,50]. The short length of miRNA targets and the lack of possibility of varying the binding sites impose certain restrictions on the design of miRNases. Therefore, when constructing miRNases, one of the tasks was to create ribonucleases exhibiting different base-specificity for the efficient degradation of miRNAs of different sequences. As a result, two types of miRNases with G-X [35,49,50] and pyrimidine-X specificity were obtained [36]. Cell culture studies have shown that engineered miRNases mediated the efficient sequence-selective cleavage of miR-21 and miR-17, leading to their down-regulation and dysfunction in tumour cells [35,36]. The high inhibitory effect of miRNases ensures their therapeutic activity in tumour cells of various histogenesis, which manifests in the 50% suppression of invasion, 40–55% inhibition of migration, 50–75% decrease in the proliferative potential of cells and the induction of apoptosis in 28% of the tumour cell population [50]. Furthermore, the most important achievement is the identification of a significant anti-tumour effect of the miRNase in a murine model of lymphosarcoma. It was found that even a single treatment of tumour cells with the developed miR-21-specific conjugate provides almost complete blocking of tumour growth in mice (Figure 3) [50]. These are the world’s first data demonstrating the use of ss-aRNase in vivo. It is worth noting that biological effects exhibited by the developed miRNases are comparable to or superior to those of currently available miR-21 inhibitors. In particular, the developed conjugates exhibit higher activity compared to the 2’-OMe miR-21-targeting antisense oligonucleotide, which provides the 50% suppression of glioblastoma cell invasion [83]; oligonucleotides containing combinations of phosphorothioate, 2’F, 2’-MOE or cEt modifications suppress tumour growth in hepatocellular carcinoma ex vivo by 40–80%, depending on the type of cells implanted in mice [84]; a commercial miR-21 inhibitor (“Ambion”, USA), promoting 50% suppression of invasion and the induction of apoptosis in 15% of the human oesophageal cancer cells [85]; and a commercial miR-21 inhibitor (“GenePharma”, China) causing a 50% suppression of proliferation, the induction of apoptosis in 25% of the pancreatic cancer cell population and 45% suppression of tumour growth in vivo [86].

As mentioned above, in addition to its own ribonuclease activity, the presence of DNA targeting oligonucleotide increases the efficiency of these inhibitors by activating intracellular RNase H. A key discovery was the detection of the synergistic action of miRNases with intracellular RNase H towards miRNA [87]. The multiple increase in the efficiency of RNA cleavage jointly by the conjugate and RNase H can be explained by: (1) the increased activity of the conjugate due to the displacement of the peptide from unproductive conformations during the interaction of RNase H with the heteroduplex; (2) an increase in the efficiency of substrate cleavage by RNase H due to the greater stabilisation of the heteroduplex with the conjugate as compared to the oligonucleotide; and (3) due to the simultaneous increase in the processivity of both the conjugate and RNase H due to the cleavage of RNA into shorter fragments and facilitation of the dissociation of the conjugate and RNase H from the complex with the target for subsequent cleavage cycles (Figure 3).

These results are of global importance, as they open up new possibilities for the use of ss-aRNases in vivo. RNase H can hold a great service even in the case of using ss-aRNases that do not exhibit a multi-turnover regime of action in vitro. We have shown that the efficiency of RNA degradation by the conjugates that do not function in a multi-turnover catalytic mode multiplies in the presence of RNase H [36,48]. When constructing miRNA-targeted “dual” conjugates, in order to preserve the ability of ss-aRNase to also recruit RNase H, we replaced adenines in the sequences of RNA recognising oligonucleotides with 2′-aminoadenines, which significantly increased the affinity of conjugate to the miRNA targets miR-21, miR-17, miR-18a and miR-155 and did not interfere with RNase H activity [48]. In the presence of RNase H, the rate of miRNA cleavage by “dual” conjugates was shown to increase 10–20-fold depending on the target sequence. Due to the simultaneous action of both ss-aRNase and RNase H, the cleavage occurred not only in the centre of miRNA (“dual” ss-aRNase) but also in its 5′- and 3′-ends (RNase H), which guarantees its complete destruction and the inhibition of functions [48]. Thus, under in vivo conditions, those ss-aRNases that maintain compatibility with RNase H may gain the advantage. In terms of clinical efficiency, the most promising ss-aRNases can be variants, the RNA recognition domain of which contains chemical modifications that simultaneously provide high hybridisation properties, nuclease resistance and compatibility with RNase H activity. Attention should be paid to recently developed modifications, such as 5′-*O*-Methylphosphonate (MEPNA) [88] or mesyl-*N*-(methanesulfonyl)-phosphoramide (mesyl or µ) [10], or gapmer constructions [89,90].

Analysis of the published data showed that highly efficient constructs of ss-aRNases have already been created (see Table 1), many of which are awaiting studies of their biomedicinal potential in vivo. In a therapeutic application, the technology of catalytic destruction of pathogenic RNAs demonstrates its validity and efficiency and offers new opportunities to design innovative therapeutic drugs.

## 5. Conclusions

### 5.1. General Principles of ss-aRNases Design

The success of ss-aRNases developed to date is encouraging; however, models employed to test the activity of ss-aRNases in vitro are maximally simplified low-component systems, using predominantly short unstructured RNAs. Biologically active RNA generally forms secondary and tertiary structures and is assembled in nucleoprotein complexes, which turn most types of intracellular RNA molecules into inaccessible to intermolecular base pairing interactions. Thus, the bioavailability of the target RNA molecule is the major challenge on the way to the development of an efficient sequence-specific enzyme. When developing therapeutic ss-aRNases, the following parameters inherent in the selected RNA target should be addressed: (1) the structural availability of RNA, namely the formation of a strong and stable secondary or tertiary structure, (2) interactions with RNA-binding proteins, and (3) localisation in an accessible and therapeutically functional cellular compartment.

The design concepts of ss-aRNases strictly depend on the structure of the target RNA, in particular, the length of the molecule, its sequence, and the secondary or tertiary structure. Similar to antisense oligonucleotides and the siRNA design, the identification of target sites in stretched structured RNA-targets for the invasion of an antisense recognition domain of ss-aRNAse is crucial for their successful application. To approach this problem, a number of sophisticated theoretical and practical methods have been proposed in order to determine effective target sites for antisense oligonucleotide cellular application, including ‘sequence-walking’, the application of random oligonucleotide libraries or genomic libraries, and microarrays [91,92]. The selection of landing sites for each specific RNA-target requires an individual approach; nevertheless, the most favourable general structural elements for binding can be emphasised, such as single-stranded areas, regions with weak intramolecular RNA/RNA base pairing, and motifs adjacent to single-stranded regions, including bulges, internal loops and dangling ends [92].

When targeting long RNA molecules, a favourable thermodynamic stability of DNA/RNA duplex over intramolecular RNA/RNA helices needs to be obtained. At this point, the binding affinity of the RNA recognition domain is also an important issue. Widely used chemically modified oligonucleotides analogues, such as 2-OMe, LNA and PNA may increase the affinity of aRNases to the RNA target. Efficient binding of the oligonucleotide domain of an ss-aRNAse induces the refolding of RNA into an alternative conformation, providing an opportunity for the catalytic group to attack the RNA-target.

Searching for the optimal length and structure of the recognition domain is of crucial importance, since the balance between the specificity and efficiency should be achieved. A reduction in the length of the binding domain to a minimum (less than 8–10 nts) can lead to (1) a decrease in binding efficiency, (2) a significant loss of specificity, and (3) an increase in off-target interactions. On the other hand, the excessive elongation of the targeting fragment can significantly reduce the efficiency of the ss-aRNase due to (1) the probability of the formation of a stable secondary structure, (2) the reduction of available complementary sites in the target molecule, and (3) the formation of an extra stable complex with the target, which cancels out the multi-cyclic catalytic mode of ss-aRNase function.

It should be noted here that the development of ss-aRNases is also associated with additional conditions. The application of these synthetic enzymes is limited, not only by the accessibility of the target site, but also by sequence requirements for their catalytic function. The motifs of sequences adjacent to ss-aRNase binding sites intended for degradation by the catalytic group should be carefully correlated with their nucleotide base specificity. The catalyst should be spatially located in close proximity to regions that are abundant in linkages that are sensitive to the corresponding type of ss-aRNase. For instance, conjugates of trisbenzimidazole and dimethylphenanthroline are efficient for adenine-rich motifs, while ss-aRNases based on short catalytic peptides predominantly cleave G-X and pyrimidine-A sites (Table 1). Interestingly, the method of peptide attachment through the C or N-terminus to an oligonucleotide can affect the specificity of cleavage: conjugates with a peptide attached through the N-terminus cleave RNA at pyrimidine-X bonds [36,47], while conjugates with a peptide attached through the C-terminus are G-X specific [35]. In addition, it is possible to vary the position of the cleavage in RNA molecules by placing the catalytic group in the central part of the oligonucleotide binding domain (“dual” and “bulge-inducing” conjugates) or close to the 5′-end of the attached oligonucleotide (“linear” conjugates).

The design of ss-aRNases targeted to short types of intracellular RNA is a special challenge as it is tightly restricted by the target sequence. The short length of RNA does not allow the position and composition of the RNase binding domain to be varied. Of particular importance is the length of the targeting oligonucleotides, which must inevitably be shorter than the target by at least several nucleotides. The introduction of additional structural elements and modifications of the oligonucleotide domain can be necessary satellites in the construction of ss-aRNases targeted to short RNAs. The implementation of a hairpin structure contributes to a significant increase in the efficiency of hybridisation due to stacking interactions [50,93]. The introduction of additional chemically modified nucleotides, such as 2′-OMe, LNA or 2-aminoadenines, or the use of a PNA backbone also significantly increases the affinity of ss-aRNses to the target.

It should be noted that the introduction of modifications can significantly change the conformational structure of ss-aRNase, leading to its deactivation. The most rational is (1) the introduction of modifications at nuclease-sensitive sites, (2) the use of modifications recruiting RNase H activity or gapmeric variant of oligonucleotides, and in some cases, and (3) the preservation of nucleotides close to the point of attachment of the catalytic group without modifications. The use of hairpin structures and chemically modified substitutions can also contribute to the nuclease resistance of conjugates, which is critical for the drug lifetime in vivo. The addition of the hairpin to the 3′-end of the oligonucleotide and attachment of the peptide to the 5′-end significantly enhances the stability of conjugates in biological media [50]. “Dual” or “bulge-inducing” conjugates require the introduction of chemical modifications into oligonucleotide shoulders to give necessary stability in physiological conditions.

Among the currently known long types of cellular RNA, disease-associated protein coding sequences, such as mRNAs, and regulatory non-coding RNAs, such as lncRNAs, circRNAs and pre-miRNAs that are located in the cytoplasm, may be attractive targets for ss-aRNases. Malfunctioning of these molecules is involved in many pathological processes during the development of a wide range of human diseases [94,95,96,97,98,99]. Nuclear RNA types (i.e., pri-miRNA) are less available for inhibition and require an additional vehicle for delivery. “Linear”, “dual” or “bulge-inducing” ss-aRNases can be successfully applied to inhibit long structured RNA targets in therapeutic purposes, while ss-aRNases with a hairpin oligonucleotide domain can be highly specific in targeting short RNA species, such as miRNAs and piRNAs, which also play a significant regulatory role in physiological and pathological processes and are strongly correlated with malignant tumour growth [87,100,101].

### 5.2. Prospects of ss-aRNases Applications

In addition, ss-aRNases may show great potential as therapeutic antibacterial agents, which are efficient in combating multidrug resistance. In addition to the direct sequence-specific degradation of bacterial coding-RNA fragments, responsible for its viability, tRNA-targeted ss-aRNases developed to date can be used to reduce the level of tmRNA in bacteria, RNA molecules that combine tRNA and mRNA elements in their structure and which are responsible for the stress resistance of bacteria and maintaining their viability [102]. In clinical applications, the inhibition of tmRNA may increase the sensitivity of bacteria to antibiotics [103].

The biomolecular platform for ss-aRNase design may also serve for the development of efficient antiviral agents that could meet the current urgent need for these drugs. Successful studies on the suppression of SARS-CoV, the virus that is highly homologous to SARS-CoV-2, by siRNAs and ribozymes [104,105,106,107], have inspired researchers to actively develop anti-SARS-CoV-2 antisense drugs. Catching up with current needs, ss-aRNases may be promising candidates for the clinical management of coronaviruses.

## Figures and Tables

**Table 1:** Design and catalytic performance of established ss-aRNases.

Catalytic Group	Linker Type	Oligonucleotide Type, Length, nts	Target Length, nts	Nucleotide Base Specificity	Cleavage Conditions, RNA: Conjugate μM	τ½ Efficiency	Ref.
Tris(2-amino- benzimidazole)	Aminohexyl	Linear DNA, 15	Synthetic RNA, 29	C-G, G-A, A-U	0.15:1.5	16.5 h	[26]
	Disulfide bridge	Linear DNA, 15, 17, 20	Synthetic RNA, 29	G-A, C-G, G-C, U-C, C-U, G-A, A-U	0.15:1.5	12.4 h/ 90% in 56 h	
	Aminohexyl	Linear Lys-PNA, 10, 15	Synthetic RNA, 29	A-U, U-C, C-U, C-G, A-G, G-A, A-A	0.15:0.75	11.2 h/ 90% in 60 h	[30]
	Aminohexyl	Lys-PNA, bulge inducing (4 nts) 11 (7-cleaver-4)	Synthetic RNA, 15	U-A, A-A, A-G	4:4 4:0.8	9 h	[31]
	Aminohexyl	Linear DNA, 15	Synthetic RNA, 22	C-A, A-A, A-U	0.15:3	14–15 h	[32]
	Aminohexyl	Linear DNA-LNA mixmers, 5’-end, 15	Synthetic RNA, 22	C-A, A-A, A-U	0.15:0.75	3.5 h	[32]
	Aminohexyl	Linear DNA- LNA mixmers, 15	Synthetic RNA ^1^, 155/412/430 *	C-A, A-A, A-U	0.25:1	2.5–3 h	[32]
	Aminohexyl	Linear DNA, 15	Synthetic RNA ^1^, 22	C-A, A-A, A-U	0.15:3	14–15 h	[71]
	Aminohexyl	Linear DNA- LNA mixmers 5′-end, 15	Synthetic RNA ^1^, 22	C-A, A-A, A-U	0.15:0.75	3.5 h	[71]
Imidazole (×24)	41 **	Linear DNA, 17	tRNA^Phe^, 76	C-A	1:10	1 h	[33]
Imidazole (×4)	41/79 **	Linear DNA, 17	tRNA^Phe^, 76	C-A	1:10	1 h	[33]
Zn(II)-2,9-dimethyl- phenanthroline	Diaminopropionic acid (Dap)	Lys-PNA, bulge inducing (4 nts) 11 (7-cleaver-4)	Synthetic RNA ^2^, 15	A-A	4:4, 4:1	11 h	[52]
	Dap	Lys-PNA, bulge inducing (3 nts) 12 (8-cleaver-4)	Synthetic RNA ^2^, 15	A-A	4:4	21 h	[52]
	Dap and additional Gly	Lys-PNA, bulge inducing (4 nts) 11 (7-cleaver-4)	Synthetic RNA ^2^, 15	A-A	4:4	12 h	[52]
	Dap and additional Gly	Lys-PNA, bulge inducing (3 nts) 12 (8-cleaver-4)	Synthetic RNA ^2^, 15	A-A	4:4	15 h	[52]
Cu(II)-2,9-dimethyl- phenanthroline	Dap	PNA, bulge inducing (4 nts) 11 (7-cleaver-4)	Synthetic RNA ^2^, 15	A-A, G-A	4:4 400:4	0.5 h	[37]
	Dap and oligoether	Lys-PNA, bulge inducing (4 nts) 11 (7-cleaver-4)	Synthetic RNA ^2^, 15	A-A	4:4	1.5 h	[38]
	Dap and additional Gly	Lys-PNA, bulge inducing (4 nts) 11 (7-cleaver-4)	Synthetic RNA ^2^, 15	A-A	4:4	3 h	[38]
Zn(II)-2,9-dimethyl- phenanthroline	Dap	Lys-PNA, bulge inducing (4 nts) 11 (7-cleaver-4)	Synthetic RNA, 15	A-A, G-A	4:4	7–8 h	[39]
	Dap	Lys-PNA, bulge inducing (3 nts) 12 (8-cleaver-4)	Synthetic RNA, 15	U-A, A-A	4:4	7–8 h	[39]
Cu(II)-2,9-dimethyl- phenanthroline	Dap	Lys-PNA, bulge inducing (4 nts) 11 (7-cleaver-4)	Synthetic RNA ^2^, 15	A-A	4:4	0.5 h	[40]
	Dap	Lys-PNA, bulge inducing (3 nts) 12 (8-cleaver-4)	Synthetic RNA ^2^, 15	U-A, A-A	4:4	14–24 h	[40]
Acridine+free Lu(III)/Zn(II)	Aminohexyl	Linear DNA, 18	Synthetic RNA, 36	C-U, U-G,	5:10	5.5–115 h	[70]
Di(Azacrown) 3-(3-hydroxypropyl) -1,5,9-triaza- cyclododecane- Zn(II)	–	Linear 2’-OMe RNA,15	Synthetic chimera 2’-***O***-Me- RNA, 19-21	C-A	18:18	90% in 120 h	[63]
Diethylenetriamine (DETA)	Polyethylene glycol (PEG)	Linear PNA, 14	Synthetic RNA, 26	G-G	2:2, 20:2	90% in 24 h	[64]
[His(Gly)_2_]-Cu(II)	PEG	Linear PNA, 14	Synthetic RNA, 26	G-A	2:2, 20:2	47.5% in 24 h	[64]
[(ArgLeu)_4_]Gly- CONH_2_	Phosphor amidate	Linear DNA, 17	tRNA^Phe^, 76	C-A, U-A	1:20	0.5 h	[34]
	Phosphor amidate	Linear DNA, 17	tRNA^Phe^, 76	C-A, U-A	1:20	0.75 h	[34]
[(ArgLeu)_2_Gly]_2_- COOH	Phosphor amidate	Linear DNA, 17	tRNA^Phe^, 76	C-A, U-A	1:20	0.9 h	[34]
[(ArgLeu)_4_]Gly	Aminohexyl and thiol-maleimide	Dual DNA, 11 + 12	tRNA^Phe^, 76	C-A, U-A	1:20	N.d.	[47]
[(ArgLeu)_2_Gly]_2_	Aminohexyl and thiol-maleimide	Dual DNA, 11 + 12	tRNA^Phe^, 76	C-A, G-X	1:20	1 h	[47]
	Aminohexyl	Bulge-inducing DNA 11-cleaver-15	tRNA^Phe^, 76	C-A, U-A, G-X	1:20	8 h	[42]
	Aminohexyl (C-termini)	Hairpin DNA, 14 * (9 bp stem)	miR-21, 22	G-X	1:20	17 ± 0.4 h/ 98% in 72 h	[35]
	Aminohexyl (C-termini)	Hairpin DNA, 16 * (6 bp stem)	miR-21, 22	G-X	1:20	83% in 72 h	
	Aminohexyl (C-termini)	Hairpin DNA, 16 * (9 bp stem)	miR-21, 22	G-X	1:20	57% in 72 h	
	Aminohexyl (C-termini)	Linear DNA, 16	miR-21, 22	G-X	1:20	15.1 ± 0.2 h/ 93 % in 72 h	
[(ArgLeu)_2_Gly]_2_	Aminohexyl (C-termini)	Hairpin DNA, 14 * (6 bp stem)	miR-21, 22	G-X	1:20	16.2 ± 0.2 h/ 99% in 72 h	[35]
					50:5	83% in 72 h	[50]
					25:5	86% in 72 h	
					10:5	87% in 72 h	
Gly(ArgLeu)_4_	(N-termini) 5′pTCAA3′ + DEG or TrEG	Hairpin DNA, 12 *	miR-21, 22	pyr-X	1:100	50% in 72 h	[36]
			miR-17, 23	pyr-X	1:20	9% in 24 h	
[(ArgLeu)_2_Gly]_2_	Aminohexyl (C-termini)	Hairpin 2′OMe + DNA, 14 * (6 bp stem)	miR-21, 22	G-X	1:20	4.9 ± 0.1 h/ 100% in 24 h	[49]
				G-X, pyr-A	10:5	77% in 72 h	
	Aminohexyl (C-termini); thiohexyl (N-termini)	Dual DNA with 2′-aminoadenines, 10 + 8	miR-17, 23	pyr-A	1:20	32% in 48 h	[48]
			miR-21, 22			30% in 48 h	
			miR-155, 23			57% in 48 h	
			miR-18a, 22			23% in 48 h	

* represents the length of region complementary to RNA target; ** represents the number of simple C–C, C–N, or P–O bonds between the 5′-terminal phosphate group of oligonucleotide B and imidazole groups of RNA-cleaving construct; ^1^ synthetic RNA corresponds to the sequence of PIM1 mRNA 3′-UTR; ^2^ synthetic RNA corresponds to the part of junction of bcr/abl mRNA. Grey colour indicates conjugates that cleave a target in a catalytic manner.

## Data Availability

Data sharing not applicable.
